# Are some populations resilient to recessions? Economic fluctuations and mortality during a period of economic decline and recovery in Finland

**DOI:** 10.1007/s10654-016-0152-8

**Published:** 2016-10-11

**Authors:** Mauricio Avendano, Heta Moustgaard, Pekka Martikainen

**Affiliations:** 1 Department of Global Health & Social Medicine, King’s College London, East Wing, Strand Campus, Strand, London, WC2R 2LS UK; 2 Department Social and Behavioral Sciences, Harvard T.H. Chan School of Public health, Boston, MA USA; 3 Population Research Unit, Department of Social Research, University of Helsinki, Helsinki, Finland; 4 Centre for Health Equity Studies (CHESS), Stockholms Universitet and Karolinska Institutet, Stockholm, Sweden; 5 The Max Planck Institute for Demographic Research, Rostock, Germany

**Keywords:** Economic recession, Unemployment, Mortality, Educational status, Registries

## Abstract

This paper uses individual-level longitudinal data on working-age Finns to examine the health effects of economic fluctuations during a period of economic decline (1989–1996) and recovery (1997–2007) in Finland. We used a nationally representative, longitudinal sample formed by linking population, employment and mortality registers (n = 698,484; 7,719,870 person-years). We implemented a region fixed-effect model that exploits within-regional variations over time in the unemployment rate to identify the effect of economic fluctuations on mortality, controlling for individual employment transitions. Unemployment rates increased from 5.2 % in 1989 to 19.8 % in 1996, declining gradually thereafter and reaching 9.7 % in 2007. Results indicate that these large fluctuations in the economy had no impact on the overall mortality of most working age Finns. The exception was highly educated men, who experienced an increase of 7 % (Rate ratio = 1.07, 95 % confidence interval 1.04, 1.10) for every one-point increase in the regional unemployment rate during the period 1989–1996 due to increased mortality from cardiovascular disease and suicide. This increase, however, was not robust in models that used the employment to population ratio as measure of the economy. Unemployment rates were unrelated to mortality among females, lower educated men, and among any group during economic recovery (1997–2007). For most Finns, we found no consistent evidence of changes in mortality in response to contractions or expansions in the economy. Possible explanations include the weak impact of the recession on wages, as well as the generous unemployment insurance and social benefit system in Finland.

**Electronic supplementary material:**

The online version of this article (doi:10.1007/s10654-016-0152-8) contains supplementary material, which is available to authorized users.

## Introduction

A growing literature suggests that job loss is associated with poorer health and higher mortality [1–6]. Some studies based on aggregate data, however, suggest that population health improves during economic contractions and deteriorates during economic expansions [7–12]. Recently, studies have started to examine this question using individual-level data and have found that there is a ‘contextual’ protective effect of economic recessions over and above the increased mortality effect of individual job loss [13, 14]. In addition, they suggest that recessions may have heterogeneous effects by increasing mortality among the unemployed while reducing mortality among the employed [14, 15].

Most studies that have used individual-level data are based in the United States, but existing evidence from other countries suggest that observed reductions in mortality during economic downturns may not hold for some European countries such as Sweden [16, 17]. A potential explanation is that social benefit programmes and benefits, which are typically more generous in the Nordic countries than the United States, might protect populations against the mortality effects of economic fluctuations. The major economic turmoil in Finland during the last two decades offers a unique opportunity to explore this question. In the late 1980s and early 1990s, Finland experienced one of the most severe recessions in post-war Europe [18]. Unemployment rates increased from 3.1 % in 1989 to 16.6 % in 1994 [19]. The depression years were followed by a period of slow but sustained economic recovery, with unemployment rates declining steadily and reaching 6.3 % in 2008, before starting to increase in 2009 (Supplementary Figure 1 in online resource) [19]. Surprisingly little research has examined how mortality changed in response to these dramatic changes in the Finnish economy [18].

This paper examines whether fluctuations in regional economic conditions were associated with changes in mortality among working-age men and women in Finland from 1989 to 2007. We exploit variations in unemployment rates as a measure of changing economic conditions across Finnish regions. While most previous studies have used aggregate mortality rates, individual-level data enables us to control for individual characteristics and examine heterogeneity in the impact of unemployment according to personal characteristics. In particular, we are able to examine whether there is a ‘contextual’ effect of economic fluctuations on mortality once changes in individual employment status are controlled for. We also examine whether the effect of economic downturns on mortality differs for individuals with different educational level. This question is motivated by evidence suggesting that the effects of recessions on labour market outcomes are born disproportionally by lower educated male workers, partly due to the demographic composition of workers across industries and occupations differentially affected by recessions [20]. Our study focuses on the years just prior to the Great Recession which included a deep economic recession (1989–1996) and subsequent years of economic recovery (1997–2007) in Finland.

## Methods

### Data and variables

Data came from a nationally representative longitudinal cohort of the Finnish population formed by linkage between continuously updated population, employment and mortality registers using unique personal identification numbers available to all permanent residents. Due to data-protection regulations, we cannot obtain data for the total population. Therefore, we obtained from Statistics Finland an 11 % random sample of the total population in 1987–2007. Regulations concerning deceased individuals are less strict, so we obtained a 80 % sample of all deaths in this period. This oversampling of deaths was performed in order to increase statistical power. To account for the differing sampling probabilities in the design, all analyses used weights provided by Statistics Finland, which gave lower weight to persons who died and higher weight to persons who survived. Our sample was restricted to individuals aged 25–65 years in order to focus on the population in the labor force. The sample comprised 698,484 persons and 7,719,870 person-years.

### Mortality

Data on mortality came from the national cause-of-death register of Statistics Finland and included dates and causes of death. Causes of death were classified into broad categories based on the 9th (1989–1995) and 10th (1996–2007) versions of the International Classification of Diseases (ICD) as follows: (a) cardiovascular disease (ICD9 codes 2891–2892, 390–4254, 4258–434, 436–4376, 4378X–444, 447–459/ICD10 codes I00–I425, I427–I99); (b) cancer (140–208, 2386, 2733/C00–C97); (c) suicide (E9500–E959/X60–X84, Y870); (d) traffic accidents (E800–E848/V01–V99); and (e) alcohol-related mortality (291, 303, 3050, 3575, 4255, 5353, 5710–5713, 5770D–5770F, 5771C–5771D, 7607A, 7795A, E860/F10, G312, G4051, G621, G721, I426, K292, K70, K860, K8600, 0354, P043, X45).

### Regional unemployment rates

As indicator of economic conditions we used the unemployment rate by region of residence and year for the period 1988–2007. Unemployment rates for age 15–74 were estimated based on our individual-level data on employment status at the end of each year. Yearly region of residence for each individual was based on 20 NUTS3-regions ranging from a few thousand to over one million inhabitants. Each region included several municipalities, but in sensitivity analysis, we found similar results when using municipalities as units of analysis. A possible concern is that some individuals live and work in different regions. However, data from Statistics Finland indicates that around 94 % of employed individuals live and work within the same region (Statistics Finland, personal communication).

### Educational level and employment status change

Educational level was classified into basic or unknown (approximately 9 years or less), secondary (12 years), lower tertiary (13–14 years), and upper tertiary (15+ years), based on the highest degree obtained. Registered employment status was measured on the last day of each year. For each year t of mortality follow-up we linked information on regional unemployment rate at the end of the previous year (t − 1) and individual-level employment status change during the previous year, i.e. from the end of year t − 2 to the end of year t − 1. Data were available since the last day of 1987, so the earliest changes in employment status were assessed between end-of-year 1987 and end-of-year 1988. We first categorized yearly employment status into employed, unemployed or out of the labor force. We then constructed a variable to indicate change in employment status between years t − 2 and t − 1, comprising six groups: (a) stable employment, if employed in both t − 2 and t − 1; (b) job loss, if employed in t − 2 but unemployed in t − 1; (c) newly employed, if unemployed in t − 2 and employed in t − 1; (d) long-term unemployed, if unemployed in both t − 2 and t − 1; and (e) out of workforce, if out of the labor force in either t − 2 or t − 1. Those with unknown employment status in year t − 2 were classified in a separate category.

### Methods of analysis

Following previous studies [9, 12, 21], we applied a region-fixed-effects model to examine how fluctuations in the regional economy were associated with short-term changes in mortality over the period 1989–2007. Supplementary Figure 2 (online resource) shows that unemployment increased from approximately 1988 to 1994 and subsequently declined in all regions in Finland, but the magnitude of these changes differed across regions. If unemployment causally influences mortality, we would expect regions that experienced larger changes in unemployment to experience larger changes in mortality rates. Our identification strategy thus relies on this regional variation in the magnitude of increases or decreases in unemployment across regions. Based on Poisson regression, our main model specification was as follows:

$$ Log(D_{ijt} ) = \beta_{0} + \beta_{1} U_{jt - 1} + \beta_{2} \left( {W_{ijt - 2} - W_{ijt - 1} } \right) + \beta_{3} A_{it - 1} + R_{jt - 1} + Z_{t} + \varepsilon_{ijt} $$

where *D* is vital status of individual *i* in region *j* at time *t*, *U* is the regional unemployment rate for region *j* at end of year *t* − 1, $W_{ijt - 2} - W_{ijt - 1}$ is the change in employment status for individual *i* in region *j* between the end of years *t* − 2 and *t* − 1, *A* represents age, $R_{jt - 1}$ is a region fixed-effect, *Z* is a year fixed-effect, and *ε* is the error term. Region fixed-effects control for time-invariant factors that differ between regions while year fixed-effects control for factors that vary uniformly across regions over time. We estimated parameters using Poisson maximum likelihood models with person-years as offset variable. Robust confidence intervals were calculated by clustering standard errors at the regional level in order to account for the interdependence of observations within regions.

**Fig. 1:**
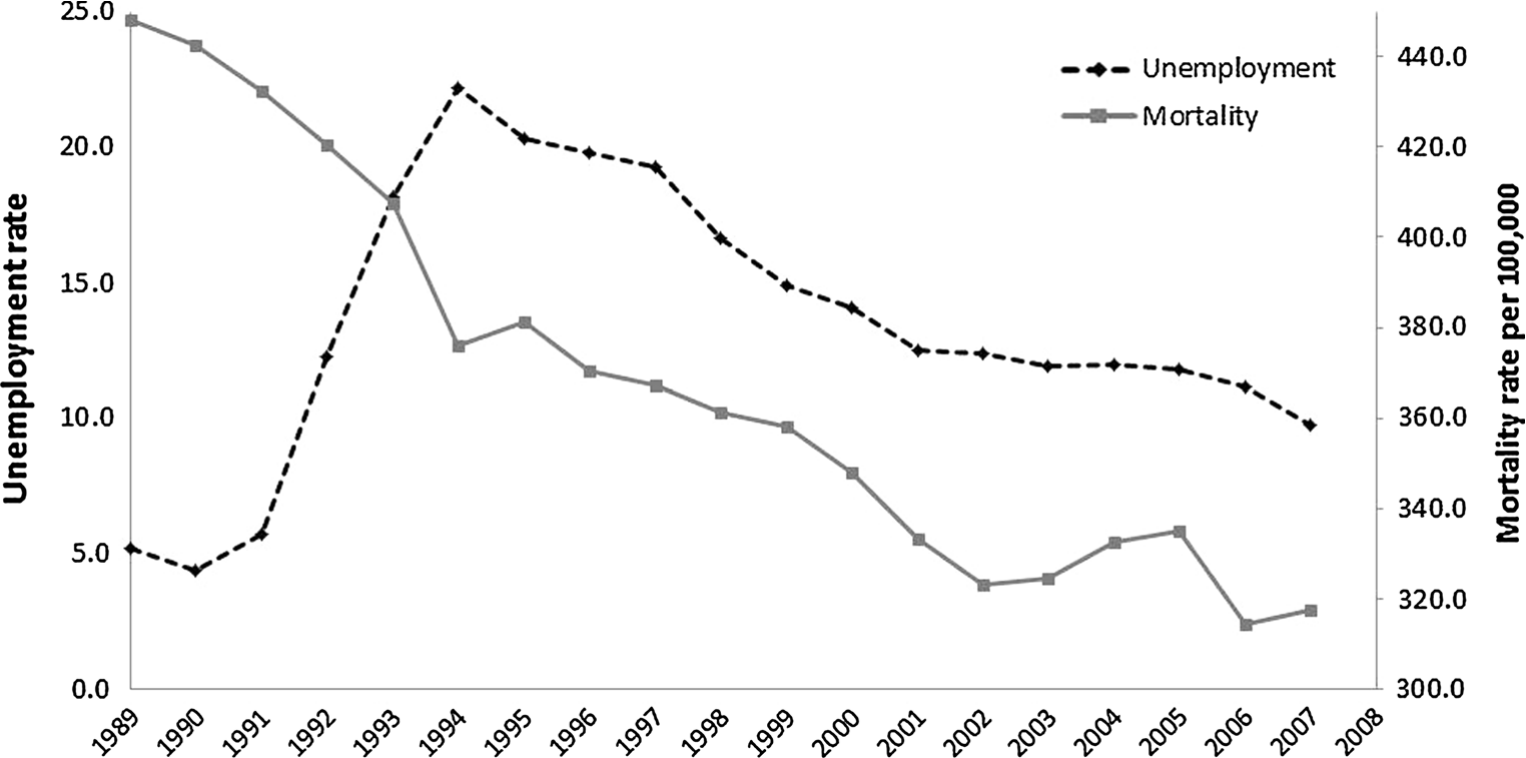
National unemployment rates (ages 15–74) and age-standardized mortality rate (ages 25–64), Finland, 1989–2007. Rates are standardized based on the 1989 Finnish population at ages 25–64 as standard

To assess educational differences in the impact of the economy on mortality, we introduced interaction terms between educational attainment and all covariates in the model. One of our key aims was to examine whether effects of fluctuations in the economy during a period of economic decline differed from those in a period of economic growth (Fig. 1). Therefore, we implemented separate models for the period 1989–1996 and 1997–2007. Analyses were conducted in STATA version 11 and incorporating weights to account for oversampling of deaths.

## Results

Table 1 shows the distribution of sample variables. Over the follow-up period, 115,671 deaths were observed in men and 48,094 in women. 65 % of all person-years corresponded to stable employment, 3 % of person-years to job loss, and 3 % to a return to work, while 23 % of the person-years were spent out of the labor force. 29 % of the sample had a tertiary education while 32 % had only basic or unknown education.

**Table 1:** Basic sample descriptives, Finland 1989–2007

	Person-years		N of deaths (unweighted)					
	Unweighted N	Weighted percentage (%)	All-cause mortality^a^	Alcohol-related	Cancer	Cardio-vascular	Suicide	Traffic accidents
Sex								
Men	4,211,465	50	115,671	14,755	23,588	37,722	10,782	2668
Women	3,508,405	50	48,094	3451	19,664	10,152	3105	914
Age								
25–49	4,566,893	67	54,348	8184	9372	9746	9516	2243
50–64	3,152,976	33	109,417	10,022	33,880	38,128	4371	1339
Employment status change								
Stable employment	4,487,791	65	42,431	3204	13,381	12,103	5201	1785
Job loss	240,801	3	4219	822	512	1002	703	167
Newly employed	192,925	3	2472	357	469	573	396	125
Long-term unemployed	488,181	6	14,685	3719	1472	3214	1729	375
Out of workforce	2,310,172	23	99,958	10,104	27,418	30,982	5858	1130
Educational level								
Upper tetriary	908,092	14	10,274	955	3609	2470	960	338
Lower tetriary	1,015,867	15	12,620	1325	4128	3135	1171	323
Secondary	2,768,682	39	48,921	6455	11,895	12,145	5822	1369
Basic or unknown	3,027,228	32	91,950	9471	23,620	30,124	5934	1552

The category ‘out of workforce’ refers to individuals that were out of the workforce in either year t − 1 or t − 2

^a^Includes 294 deaths with unknown cause

Table 2 summarizes estimates from the region and year fixed-effect models for men in periods 1989–1996 (column 1) and 1997–2007 (column 3). At the individual level, we found that the transition to unemployment was associated with a doubling of the mortality risk relative to those who remained employed, while those who regained employment had a higher risk of dying than those continually employed. Those in long-term unemployment and out of the workforce had also larger mortality than those with stable employment. The relationship between individual employment transitions and mortality was very similar across periods. Controlling for individual employment transitions, a change in regional unemployment rate was unrelated to mortality for both men and women. Supplementary Figure 3 (online resource) summarizes estimates for the unemployment rate for five broad causes of death. There was no association between unemployment rates and any cause of death for men. Among women, mortality from cancer (RR 1.02, 95 % CI 1.01, 1.03) increased when unemployment rose in the period 1989–1996, but there was no association for other causes of death. In models that used the full period from 1989 to 2007 and incorporated interaction terms between each variable and a period indicator (results not shown), we found no evidence of significant differences in the impact of unemployment rates on mortality between the two periods (*p* > .05).

**Table 2:** Regional unemployment rate, employment status change and all-cause mortality, Finland, 1989–1996 and 1997–2007

	1989–1996				1997–2007			
	Males		Females		Males		Females	
	RR	95 % CI	RR	95 % CI	RR	95 % CI	RR	95 % CI
Unemployment rate	0.99	(0.98, 1.01)	1.01	(1.00, 1.03)	1.01	(0.99, 1.02)	1.00	(0.99, 1.02)
Age	1.05	(1.05, 1.06)	1.06	(1.06, 1.06)	1.05	(1.05, 1.06)	1.06	(1.05, 1.06)
Employm. status change								
Stable employment	1.00		1.00		1.00		1.00	
Job loss	2.10	(1.97, 2.24)	1.51	(1.21, 1.89)	2.50	(2.27, 2.76)	1.70	(1.50, 1.93)
Newly employed	1.89	(1.78, 2.01)	1.23	(1.10, 1.38)	1.89	(1.76, 2.03)	1.34	(1.22, 1.47)
Long-term unemployed	3.36	(3.13, 3.60)	2.19	(1.90, 2.53)	4.40	(4.08, 4.74)	2.67	(2.32, 3.06)
Out of workforce	4.37	(4.19, 4.57)	3.77	(3.67, 3.87)	5.53	(5.38, 5.68)	5.00	(4.72, 5.29)

All models include region and year fixed effects, but estimates are omitted from Table

The category ‘out of workforce’ refers to individuals that were out of the workforce in either year t − 1 or t − 2

**Fig. 2:**
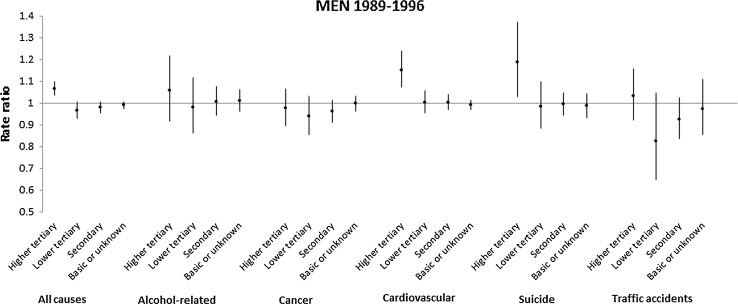
Impact of one-point increase in the regional unemployment rate on cause-specific mortality, Finland, males, 1989–1996. All models include age, employment status change, region and year fixed-effects, as well as interactions of educational level with regional unemployment rates, age, employment status change, region and year

Table 3 shows estimates from a model that incorporates interactions of educational level with all individual and regional-level variables. For brevity, we only present interaction terms with regional unemployment rate. Results reveal some heterogeneity by educational level during the period of deep economic recession (1989–1996) among men. In particular, a one-point increase in the unemployment rate was associated with a 7 % (RR 1.07, 95 % CI 1.04, 1.10) increase in all-cause mortality for men with tertiary education, while there was no association for less educated men, or for any educational group among women. Figure 2 disaggregates results for the period 1989–1996 for specific causes of death among men. Estimates suggest that the increase in mortality for highly educated men during years of rising unemployment was driven by an increase in mortality from cardiovascular disease and suicide.

**Table 3:** Interaction between educational level and regional unemployment rates, employment status change and all-cause mortality, Finland, 1989–1996 and 1997–2007

	1989–1996				1997–2007			
	Males		Females		Males		Females	
	RR	95 % CI	RR	95 % CI	RR	95 % CI	RR	95 % CI
Unemployment rate	1.07	(1.04, 1.10)	0.99	(0.91, 1.07)	0.98	(0.94, 1.02)	1.04	(0.99, 1.09)
Educational level								
Upper tertiary	1.00		1.00		1.00		1.00	
Lower tertiary	2.39	(1.78, 3.21)	0.79	(0.50, 1.24)	2.14	(1.41, 3.25)	2.18	(1.43, 3.34)
Secondary	5.77	(4.57, 7.29)	1.87	(1.22, 2.88)	3.18	(2.38, 4.26)	4.30	(3.05, 6.05)
Basic or unknown	14.18	(11.25, 17.88)	7.98	(5.50, 11.58)	11.07	(7.72, 15.88)	22.52	(12.74, 39.81)
Lower tertiary*unemp. rate	0.91	(0.87, 0.95)	1.03	(0.93, 1.14)	1.04	(0.98, 1.10)	0.97	(0.90, 1.04)
Secondary*unemp. rate	0.92	(0.88, 0.96)	1.03	(0.95, 1.13)	1.03	(0.99, 1.06)	0.97	(0.92, 1.02)
Basic/unknown*unemp. rate	0.93	(0.90, 0.96)	1.03	(0.95, 1.12)	1.03	(0.99, 1.08)	0.97	(0.92, 1.02)

All models include age, employment status change, region, year fixed-effects, and interactions of educational level with all variables

### Sensitivity analyses

We carried out analyses to examine whether unemployment rates up to 6 years earlier had a lagged effect on mortality (Supplementary Table 1, in online resource). We found no evidence that lagged unemployment rates increased overall mortality in either period. In a model that examined interactions with education, we found that an increase in unemployment rates in year t − 2 during the deep economic recession (1989–1996) was associated with an increase in mortality among highly educated men (RR 1.05, 95 % CI 1.01, 1.09), but this effect was not observed for years t − 3 to t − 6 (Supplementary Table 2, in online resource).

A possible bias may arise if regional unemployment rates are correlated with regional trends in other factors that affect mortality. We therefore replicated all models with detrended regional unemployment rates using a Hodrick–Prescott Filter (HP) [22], which separates the cyclical component of a time-series from its general trend by estimating the annual deviation from a smoothed trend curve, thus controlling for regional linear trends. We used a smoothing parameter of 100, but results did not change with alternative parameters. Results from all models using this detrended version of unemployment rates are presented in Supplementary Figures 4–6 (online resource). Results were very similar to those observed using crude unemployment rates, with one exception. For the years 1989–1996, increased unemployment was associated with decreased all-cause mortality among men when using detrended unemployment rates (RR 0.98, 95 % CI 0.97, 0.99) (Supplementary Figure 4, online resource). This effect among men was driven by a decline in mortality from traffic accidents (RR 0.92, 95 % CI 0.83, 1.01) and cancer (RR 0.97, 95 % CI 0.93, 1.01) when unemployment rates rose, although these declines were not significant. Supplementary Figure 5 (online resource) shows that the interaction between unemployment rate and educational level remains significant in detrended models, with highly educated men suffering disproportionately from increased mortality when unemployment rises.

To control for secular declines in mortality throughout all regions (Fig. 1), our models included year fixed effects. However, there maybe region-specific mortality trends not accounted for by year fixed effects. To examine this, we replicated models incorporating regional linear trends in addition to year fixed effects (Supplementary Table 3, in online resource). Results from these models were very similar to those in our original specification in Table 2.

We carried out sensitivity analyses that incorporated household income in the model. We used data from household taxable income at time t − 1, which comprised gross income of all household members, including wages, capital income, and taxable income transfers, but excluded tax-exempted social benefits, adjusting for inflation [23] and household structure using the OECD scale [24]. Results incorporating the log of household income in the models are summarized in Supplementary Table 4 (online resource). As expected, higher household income was associated with lower mortality among both men and women. However, controlling for income had no impact on the association between unemployment rates and mortality. In addition, countercyclical mortality among tertiary educated men in the recession period was robust to controlling for income (results not shown).

While unemployment rates might best reflect the impact of the recession in the early 1990s, we carried out sensitivity analyses using the employment to population ratio, an alternative labour market measure that refers to the number of employed individuals divided by the size of the working-age population (those aged 15–64) in each region. Results are summarized in Supplementary Tables 5 and 6 (online resource). Overall, for the majority of groups, we find no association between employment to population ratios and mortality. However, among males during the 1989–1996 period of recession, an increase in the regional employment to population ratio–which signals an improvement in the regional economy-was associated with an increase in the regional mortality rate (IRR 1.04, 95 % CI 1.03–1.05), an effect driven by men in the two lowest levels of education (basic and secondary). In all other groups and periods, regional employment rates had no effects on mortality.

A final consideration in our analysis are the self-employed, who cannot be distinguished from employed individuals in our yearly data. In sensitivity analysis, however, we incorporated self-employment data available from 5-year censuses in 1985, 1990, 1995, 2000, and 2005, extrapolating information for the inter-census years based on the latest census. We found that self-employed men had a 15–20 % higher all-cause mortality compared with the employed in both periods, however, controlling for self-employment had no impact on our estimates of the effects of regional unemployment rates on mortality. Among women, self-employment was not associated with mortality. In addition, the increasing mortality for tertiary educated men in the recession period was robust to controlling for self-employment.

## Discussion

Based on individual-level registry data, our study suggests that, for most Finns, there is no independent ‘contextual’ effect of economic downturns on mortality. Higher educated men experienced increased mortality when unemployment rose during the deep economic recession of the early 1990s, due to increased mortality from CVD and suicide. However, this finding was not replicated when using the employment to population ratio as a measure of economic conditions. We found no consistent evidence that economic fluctuations influence female mortality.

### Are Finns resilient to economic recessions?

The most remarkable finding in our study is that, unlike the United States and some other OECD countries [7–12, 25, 26], we found no evidence that an increase in regional unemployment rates is associated with a decline in the regional mortality rate. This is true even for the deep recession in 1989–1996. Our results are partly in line with other studies from Scandinavian countries that have failed to replicate a decline in mortality during recessions. Based on individual-level data, a study in Sweden found that overall mortality increased during economic recessions among men, while there was no effect among women [16]. Another study in Sweden found that recessions were associated with increased incidence and mortality from acute myocardial infarction among prime-working age Swedish men [17]. Recent evidence also suggests that the well-documented relationship between business cycles and mortality for the US [8, 9, 21] may not hold for more recent periods [27]. Based on data from 1979 to 2009, Ruhm found that the impact of recessions on some causes of death reported in earlier work [8, 9, 21, 28–30] shifted and became null using more recent data [31]. Overall, these findings suggest that the relationship between business cycles and mortality may be period and context specific [32].

There are at least two potential social policy mechanisms that may explain the resilience of Finns to economic fluctuations. The first mechanism refers to the limited impact of the recession on the incomes of Finns, often attributed to the large role of unions in collective agreements related to nominal wages. Union density in Finland was 72 % in 1988–1994, but collective agreements covered over 95 % of the workforce [33, 34]. Since unions have to agree on wage cuts suggested by employers [35, 36], Finnish workers have a strategic advantage in wage negotiations. As a result, nominal wages were frozen by the collective agreements over the period 1992–1993; the rate of inflation was slower than expected and there was a continuation of a small but positive wage drift. This means that for most Finns aggregate real wages remained largely unchanged in 1992–1994 [35], which may explain the weak effect of increasing unemployment on mortality.

Wage changes regulated by unions, however, only benefitted job stayers, while most of the burden of the recession fell on workers who lost their jobs. We found, however, no independent effect of rising unemployment rates on the mortality of displaced workers. A possible explanation comes from the generous unemployment benefit system in Finland, which is particularly generous in terms of replacement rates and duration of compensation relative to other EU countries [37]. During 1983–1994, Finland combined a system of high unemployment benefit replacement rates–approximately 63 % for a period of 2 years—with relatively late referral to labour market activation [38]. Finnish workers were thus protected against large income losses from unemployment during the recession, and as a result, Finnish households suffered economic losses that were smaller than those predicted based on the decline in GDP [38]. This hypothesis is supported by recent evidence suggesting that generous unemployment benefits can offset the impact of increasing unemployment rates on suicide [39], as well as the impact of job loss on self-rated health [40].

We found some evidence that the deep economic recession increased mortality from suicide and CVD for highly educated Finnish men. We note that this increase is not only driven by job loss as we controlled for individual employment transitions. Several studies have shown that suicides increase when the economy contracts [9, 39, 41–44]. These studies often interpret this effect as the result of economic strain arising from unemployment particularly for economically constrained families in lower socioeconomic groups [41, 42, 45], but it is not clear how this would apply to highly educated men. A possible explanation is that, in the context of generous unemployment benefits for economically constrained workers, recessions increase psychosocial stress particularly among highly educated workers, due to their higher career expectations, the potentially larger income losses and scaring effects of job loss for their future careers and long-term economic well-being [46]. Likewise, evidence suggests a link between psychosocial stress and mortality from cardiovascular disease, particularly for ischemic heart disease [47–51], for which we observed a particularly marked increased in response to increasing unemployment. Nevertheless, the increase in mortality among highly educated men should be interpreted with caution, as it was not replicated when using the employment to population ratio as measure of the economy.

### Limitations

Despite several strengths, some limitations should be considered in our study. We identified the impact of the recession out of variations in the change in unemployment rates across Finnish regions. A potential concern is that all regions experienced similar increases in unemployment as a result of the crisis, and there was therefore little difference between ‘treated’ and ‘control’ regions. On the other hand, while all regions indeed experienced rising unemployment, the size of this increase ranged from 13 % points in the region of Varsinais-Suomi to 17.7 % points in Päijät–Häme, a difference of 4.7 % points (Supplementary Figure 2, online resource). Nevertheless, we note that our study compares regions all of which experienced rising unemployment, an approach that may underestimate the impact of economic downturns on mortality.

Our study focused on the period from 1989 to 2007 and did not cover the more recent increases in unemployment following the great recession. We restricted the analysis to this period because our data were only harmonized up to 2007. In addition, our intention was to focus on the period of large increasing unemployment in Finland that occurred in the early 1990s, and the subsequent gradual decline in unemployment that ended in 2008, and after which unemployment started to increase again (Supplementary Figure 1, online resource). While examining this period would be of interest, it is unlikely that our results would change much by incorporating the more recent period given the relatively smaller fluctuations in unemployment rates.

We were not able to distinguish involuntary job loss due to business closure or mass lay-offs from unemployment due to health or other reasons. Estimates for individual unemployment, therefore, cannot be causally interpreted as they combine both the potential causal effect of unemployment with the impact of reverse causation or omitted confounding variables [32]. Nevertheless, our focus was on the impact of regional unemployment rates, a measure of the business cycle that is not correlated with individual characteristics. The assumption of this approach is that short-term fluctuations in unemployment rates are primarily driven by changes in economic output and have less to do with changes in the health composition of regions [7–12, 25, 26], which we have no reason to believe changed in the aggregate during the period of study, e.g., there was no major epidemic or sudden event that would have systematically impoverished the health of regions and lowered local economic productivity. Based on this rationale, we believe our estimates capture the impact of presumably exogenous changes in the regional economy on the health of individuals in each region.

## Conclusion

The large increase in unemployment during the early 1990s and subsequent economic growth had little impact on the mortality of working-age men and women in Finland. These findings contradict results from some studies that have found declines in mortality during periods of increasing unemployment. We speculate that weak effects of economic fluctuations on wages, paired with a system of generous unemployment and other social benefits, may have been effective in buffering the impact of the economic collapse on the mortality of Finns.

## Electronic supplementary material

Below is the link to the electronic supplementary material.

Supplementary material 1 (DOCX 1105 kb)
