# Supplementary material for: Are some populations resilient to recessions? Economic fluctuations and mortality during a period of economic decline and recovery in Finland
Source: Eur J Epidemiol. 2016 Oct 11;32(1):77–85. doi: 10.1007/s10654-016-0152-8 (PMC5331077; doi:10.1007/s10654-016-0152-8)
Supplement: Supplementary file 1 — Supplementary material 1 (DOCX 1105 kb) [file 10654_2016_152_MOESM1_ESM.docx]

**ONLINE SUPPLEMENTARY MATERIAL**

**Are some populations resilient to recessions? Economic fluctuations and mortality during a period of economic decline and recovery in Finland**

**Authors:** Mauricio Avendano(*)^1,2^ **,** Heta Moustgaard^3^, Pekka Martikainen^3,4,5^

**Affiliations:**

1. Department of Social Science, Health and Medicine, King’s College London, London, UK
2. Department Social and Behavioral Sciences, Harvard School of Public Health, Boston, USA
3. Population Research Unit, Department of Social Research, University of Helsinki, Helsinki, Finland
4. Centre for Health Equity Studies (CHESS), Stockholms Universitet and Karolinska Institutet, Sweden
5. The Max Planck Institute for Demographic Research, Germany

**Address for correspondence:**

Mauricio Avendano, PhD

Department of Social Science, Health and Medicine

East Wing, Strand Campus, King's College, London, Strand

London WC2R 2LS, United Kingdom

Email: [mauricio.avenano_pabon@kcl.ac.uk](mailto:mauricio.avenano_pabon@kcl.ac.uk)

Tel. +44 7 421 994 782

**Supplementary Table 1. Lagged effect of regional unemployment rates on all-cause mortality, Finland, 1989-1996 & 1997-2007**

|  | 1989-1996 | | | | | | | | | | | | 1997-2007 | | | | | | | | | | |  |
| --- | --- | --- | --- | --- | --- | --- | --- | --- | --- | --- | --- | --- | --- | --- | --- | --- | --- | --- | --- | --- | --- | --- | --- | --- |
|  | Males | | | | | | Females | | | | | | Males | | | | | | Females | | | | | |
|  | RR |  | 95%CI | | |  | RR |  | 95%CI | | |  | RR |  | 95%CI | | |  | RR |  | 95%CI | | |  |
|  |  |  |  |  |  |  |  |  |  |  |  |  |  |  |  |  |  |  |  |  |  |  |  |  |
| Unemployment rate t-1 | 1.00 | ( | 0.98 | , | 1.02 | ) | 1.00 | ( | 0.98 | , | 1.03 | ) | 1.01 | ( | 0.99 | , | 1.02 | ) | 1.01 | ( | 0.99 | , | 1.03 | ) |
| Unemployment rate t-2 | 1.00 | ( | 0.98 | , | 1.02 | ) | 1.01 | ( | 0.99 | , | 1.03 | ) | 0.99 | ( | 0.98 | , | 1.00 | ) | 1.00 | ( | 0.98 | , | 1.02 | ) |
| Unemployment rate t-3 | 1.01 | ( | 0.99 | , | 1.03 | ) | 0.98 | ( | 0.95 | , | 1.01 | ) | 0.99 | ( | 0.97 | , | 1.00 | ) | 0.99 | ( | 0.98 | , | 1.01 | ) |
| Unemployment rate t-4 | 1.00 | ( | 0.96 | , | 1.03 | ) | 0.99 | ( | 0.95 | , | 1.04 | ) | 0.99 | ( | 0.97 | , | 1.01 | ) | 1.00 | ( | 0.98 | , | 1.02 | ) |
| Unemployment rate t-5 | 1.01 | ( | 0.97 | , | 1.05 | ) | 0.97 | ( | 0.89 | , | 1.06 | ) | 0.99 | ( | 0.98 | , | 1.01 | ) | 1.00 | ( | 0.98 | , | 1.01 | ) |
|  |  |  |  |  |  |  |  |  |  |  |  |  |  |  |  |  |  |  |  |  |  |  |  |  |
|  |  |  |  |  |  |  |  |  |  |  |  |  |  |  |  |  |  |  |  |  |  |  |  |  |
| *All models include age, employment status change, region and year fixed effects but estimates are omitted from Table. All covariates are measured simultaneously with regional unemployment rate* | | | | | | | | | | | | | | | | | | | | | | | | |
| *The category 'out of workforce' refers to individuals that were out of the workforce in either year* | | | | | | | | | | | | | | | | |  |  |  |  |  |  |  |  |

**Supplementary Table 2. Interaction between educational level and lagged regional unemployment rates and all-cause mortality, Finland, 1989-1996 & 1997-2007**

|  | 1989-1996 | | | | | | | | | | | | |  | 1997-2007 | | | | | | | | | | | |  |
| --- | --- | --- | --- | --- | --- | --- | --- | --- | --- | --- | --- | --- | --- | --- | --- | --- | --- | --- | --- | --- | --- | --- | --- | --- | --- | --- | --- |
|  | Males | | | | | |  | Females | | | | | |  | Males | | | | | |  | Females | | | | | |
|  | RR |  | 95%CI | | |  |  | RR |  | 95%CI | | |  |  | RR |  | 95%CI | | |  |  | RR |  | 95%CI | | |  |
| **Year t-2** |  |  |  |  |  |  |  |  |  |  |  |  |  |  |  |  |  |  |  |  |  |  |  |  |  |  |  |
| Unemployment rate | 1.05 | ( | 1.01 | , | 1.09 | ) |  | 0.99 | ( | 0.92 | , | 1.07 | ) |  | 1.00 | ( | 0.96 | , | 1.03 | ) |  | 0.99 | ( | 0.93 | , | 1.05 | ) |
| Lower tetriary*unemp. rate | 0.94 | ( | 0.91 | , | 0.97 | ) |  | 0.97 | ( | 0.88 | , | 1.06 | ) |  | 1.02 | ( | 0.97 | , | 1.07 | ) |  | 0.98 | ( | 0.91 | , | 1.06 | ) |
| Secondary* unemp.rate | 0.96 | ( | 0.91 | , | 1.01 | ) |  | 1.05 | ( | 0.98 | , | 1.13 | ) |  | 1.01 | ( | 0.98 | , | 1.04 | ) |  | 1.05 | ( | 0.98 | , | 1.12 | ) |
| Basic/unknown* unemp.rate | 0.96 | ( | 0.92 | , | 1.00 | ) |  | 1.01 | ( | 0.93 | , | 1.09 | ) |  | 1.01 | ( | 0.98 | , | 1.05 | ) |  | 1.03 | ( | 0.97 | , | 1.09 | ) |
| **Year t-3** |  |  |  |  |  |  |  |  |  |  |  |  |  |  |  |  |  |  |  |  |  |  |  |  |  |  |  |
| Unemployment rate | 1.01 | ( | 0.96 | , | 1.07 | ) |  | 0.99 | ( | 0.93 | , | 1.05 | ) |  | 1.00 | ( | 0.97 | , | 1.04 | ) |  | 0.92 | ( | 0.86 | , | 0.98 | ) |
| Lower tetriary* unemp.rate | 1.02 | ( | 0.93 | , | 1.10 | ) |  | 0.98 | ( | 0.90 | , | 1.06 | ) |  | 1.00 | ( | 0.96 | , | 1.05 | ) |  | 1.07 | ( | 0.98 | , | 1.18 | ) |
| Secondary* unemp.rate | 0.99 | ( | 0.93 | , | 1.06 | ) |  | 1.00 | ( | 0.93 | , | 1.07 | ) |  | 0.98 | ( | 0.94 | , | 1.02 | ) |  | 1.10 | ( | 1.04 | , | 1.17 | ) |
| Basic/unknown* unemp. rate | 0.98 | ( | 0.92 | , | 1.05 | ) |  | 1.04 | ( | 0.97 | , | 1.12 | ) |  | 1.00 | ( | 0.95 | , | 1.04 | ) |  | 1.09 | ( | 1.02 | , | 1.17 | ) |
| **Year t-4** |  |  |  |  |  |  |  |  |  |  |  |  |  |  |  |  |  |  |  |  |  |  |  |  |  |  |  |
| Unemployment rate | 0.99 | ( | 0.92 | , | 1.07 | ) |  | 0.93 | ( | 0.83 | , | 1.04 | ) |  | 0.99 | ( | 0.95 | , | 1.03 | ) |  | 0.95 | ( | 0.90 | , | 1.01 | ) |
| Lower tetriary* unemp.rate | 1.02 | ( | 0.90 | , | 1.17 | ) |  | 1.03 | ( | 0.85 | , | 1.26 | ) |  | 0.99 | ( | 0.96 | , | 1.03 | ) |  | 1.04 | ( | 0.95 | , | 1.13 | ) |
| Secondary* unemp.rate | 1.02 | ( | 0.95 | , | 1.10 | ) |  | 1.06 | ( | 0.96 | , | 1.18 | ) |  | 1.00 | ( | 0.95 | , | 1.04 | ) |  | 1.05 | ( | 0.98 | , | 1.13 | ) |
| Basic/unknown* unemp.rate | 1.03 | ( | 0.94 | , | 1.12 | ) |  | 1.06 | ( | 0.94 | , | 1.20 | ) |  | 1.01 | ( | 0.97 | , | 1.04 | ) |  | 1.05 | ( | 0.98 | , | 1.12 | ) |
| **Year t-5** |  |  |  |  |  |  |  |  |  |  |  |  |  |  |  |  |  |  |  |  |  |  |  |  |  |  |  |
| Unemployment rate | 0.95 | ( | 0.83 | , | 1.10 | ) |  | 1.18 | ( | 1.00 | , | 1.40 | ) |  | 0.98 | ( | 0.93 | , | 1.05 | ) |  | 0.96 | ( | 0.91 | , | 1.02 | ) |
| Lower tetriary* unemp.rate | 1.08 | ( | 0.85 | , | 1.37 | ) |  | 0.85 | ( | 0.70 | , | 1.04 | ) |  | 1.00 | ( | 0.95 | , | 1.06 | ) |  | 1.05 | ( | 0.95 | , | 1.16 | ) |
| Secondary* unemp.rate | 1.02 | ( | 0.88 | , | 1.18 | ) |  | 0.78 | ( | 0.65 | , | 0.93 | ) |  | 1.00 | ( | 0.94 | , | 1.06 | ) |  | 1.04 | ( | 0.98 | , | 1.10 | ) |
| Basic/unknown* unemp. rate | 1.06 | ( | 0.90 | , | 1.25 | ) |  | 0.86 | ( | 0.71 | , | 1.03 | ) |  | 1.01 | ( | 0.95 | , | 1.07 | ) |  | 1.04 | ( | 0.97 | , | 1.11 | ) |
| **Year t-6** |  |  |  |  |  |  |  |  |  |  |  |  |  |  |  |  |  |  |  |  |  |  |  |  |  |  |  |
| Unemployment rate | 0.94 | ( | 0.75 | , | 1.18 | ) |  | 0.76 | ( | 0.58 | , | 1.01 | ) |  | 0.97 | ( | 0.92 | , | 1.03 | ) |  | 1.00 | ( | 0.95 | , | 1.05 | ) |
| Lower tetriary* unemp. rate | 1.15 | ( | 0.86 | , | 1.54 | ) |  | 1.22 | ( | 0.87 | , | 1.71 | ) |  | 1.02 | ( | 0.97 | , | 1.07 | ) |  | 1.00 | ( | 0.91 | , | 1.09 | ) |
| Secondary*unemp. rate | 1.02 | ( | 0.78 | , | 1.32 | ) |  | 1.36 | ( | 1.03 | , | 1.80 | ) |  | 1.02 | ( | 0.96 | , | 1.08 | ) |  | 1.00 | ( | 0.95 | , | 1.05 | ) |
| Basic/unknown* unemp. rate | 1.09 | ( | 0.87 | , | 1.36 | ) |  | 1.27 | ( | 0.97 | , | 1.67 | ) |  | 1.03 | ( | 0.97 | , | 1.08 | ) |  | 0.99 | ( | 0.94 | , | 1.04 | ) |
| *All models include age, employment status change, region and year fixed effects but estimates are omitted from Table. All covariates are measured simultaneously with regional unemployment rate.* | | | | | | | | | | | | | | | | | | | | | | | | | | | |
| *The category 'out of workforce' refers to individuals that were out of the workforce in either year* | | | | | | | | | | | | | | | |  |  |  |  |  |  |  |  |  |  |  |  |

**Supplementary Table 3. Regional unemployment rates, employment status change and all-cause mortality (model with regional linear trends), Finland, 1989-1996 & 1997-2007**

|  | 1989-1996 | | | | | | | | | | | | 1997-2007 | | | | | | | | | | | |
| --- | --- | --- | --- | --- | --- | --- | --- | --- | --- | --- | --- | --- | --- | --- | --- | --- | --- | --- | --- | --- | --- | --- | --- | --- |
|  | Males | | | | | | Females | | | | | | Males | | | | | | Females | | | | | |
|  | RR |  | 95%CI | | |  | RR |  | 95%CI | | |  | RR |  | 95%CI | | |  | RR |  | 95%CI | | |  |
|  |  |  |  |  |  |  |  |  |  |  |  |  |  |  |  |  |  |  |  |  |  |  |  |  |
| Unemployment rate | 0.99 | ( | 0.97 | , | 1.01 | ) | 1.01 | ( | 0.98 | , | 1.04 | ) | 1.01 | ( | 0.99 | , | 1.03 | ) | 1.01 | ( | 0.98 | , | 1.04 | ) |
| Employm. status change |  |  |  |  |  |  |  |  |  |  |  |  |  |  |  |  |  |  |  |  |  |  |  |  |
| *Stable employment* | 1.00 |  |  |  |  |  | 1.00 |  |  |  |  |  | 1.00 |  |  |  |  |  | 1.00 |  |  |  |  |  |
| *Job loss* | 2.14 | ( | 2.01 | , | 2.29 | ) | 1.56 | ( | 1.25 | , | 1.96 | ) | 2.52 | ( | 2.29 | , | 2.77 | ) | 1.74 | ( | 1.54 | , | 1.96 | ) |
| *Newly employed* | 1.92 | ( | 1.81 | , | 2.04 | ) | 1.26 | ( | 1.13 | , | 1.41 | ) | 1.92 | ( | 1.78 | , | 2.07 | ) | 1.37 | ( | 1.26 | , | 1.50 | ) |
| *Long-term unemployed* | 3.39 | ( | 3.16 | , | 3.65 | ) | 2.25 | ( | 1.96 | , | 2.58 | ) | 4.39 | ( | 4.09 | , | 4.72 | ) | 2.73 | ( | 2.38 | , | 3.13 | ) |
| *Out of workforce* | 4.87 | ( | 4.63 | , | 5.12 | ) | 4.56 | ( | 4.42 | , | 4.70 | ) | 6.33 | ( | 4.09 | , | 6.53 | ) | 6.57 | ( | 6.19 | , | 6.97 | ) |
|  |  |  |  |  |  |  |  |  |  |  |  |  |  |  |  |  |  |  |  |  |  |  |  |  |
| *All models include age, region fixed effects, year fixed effects, and regional linear trends, but estimates are omitted from Table*  *The category 'out of workforce' refers to individuals that were out of the workforce in either year t-1 or t-2* | | | | | | | | | | | | | | | | | | | | | | | | |

**Supplementary Table 4. Regional unemployment rates, employment status change and all-cause mortality (controlling for household income), Finland, 1989-1996 & 1997-2007**

|  | 1989-1996 | | | | | | | | | | | | 1997-2007 | | | | | | | | | | | |
| --- | --- | --- | --- | --- | --- | --- | --- | --- | --- | --- | --- | --- | --- | --- | --- | --- | --- | --- | --- | --- | --- | --- | --- | --- |
|  | Males | | | | | | Females | | | | | | Males | | | | | | Females | | | | | |
|  | RR |  | 95%CI | | |  | RR |  | 95%CI | | |  | RR |  | 95%CI | | |  | RR |  | 95%CI | | |  |
|  |  |  |  |  |  |  |  |  |  |  |  |  |  |  |  |  |  |  |  |  |  |  |  |  |
| Unemployment rate | 0.99 | ( | 0.98 | , | 1.01 | ) | 1.01 | ( | 1.00 | , | 1.03 | ) | 1.00 | ( | 0.99 | , | 1.02 | ) | 1.00 | ( | 0.99 | , | 1.02 | ) |
| Employm. status change |  |  |  |  |  |  |  |  |  |  |  |  |  |  |  |  |  |  |  |  |  |  |  |  |
| *Stable employment* | 1.00 |  |  |  |  |  | 1.00 |  |  |  |  |  | 1.00 |  |  |  |  |  |  |  |  |  |  |  |
| *Job loss* | 2.01 | ( | 1.91 | , | 2.13 | ) | 1.45 | ( | 1.16 | , | 1.81 | ) | 2.37 | ( | 2.17 | , | 2.60 | ) | 1.60 | ( | 1.41 | , | 1.82 | ) |
| *Newly employed* | 1.81 | ( | 1.69 | , | 1.94 | ) | 1.18 | ( | 1.06 | , | 1.33 | ) | 1.80 | ( | 1.67 | , | 1.95 | ) | 1.27 | ( | 1.16 | , | 1.40 | ) |
| *Long-term unemployed* | 3.06 | ( | 2.90 | , | 3.23 | ) | 2.03 | ( | 1.77 | , | 2.32 | ) | 3.93 | ( | 3.68 | , | 4.20 | ) | 2.37 | ( | 2.08 | , | 2.71 | ) |
| *Out of workforce* | 3.90 | ( | 3.80 | , | 4.00 | ) | 3.34 | ( | 3.25 | , | 3.42 | ) | 4.83 | ( | 4.70 | , | 4.96 | ) | 4.24 | ( | 4.03 | , | 4.46 | ) |
| Log household income | 0.91 | ( | 0.90 | , | 0.91 | ) | 0.87 | ( | 0.85 | , | 0.89 | ) | 0.91 | ( | 0.90 | , | 0.93 | ) | 0.87 | ( | 0.84 | , | 0.89 | ) |
|  |  |  |  |  |  |  |  |  |  |  |  |  |  |  |  |  |  |  |  |  |  |  |  |  |
| *All models include age, region and year fixed effects but estimates are omitted from Table*  *The category 'out of workforce' refers to individuals that were out of the workforce in either year t-1 or t-2* | | | | | | | | | | | | | | | | | | | | | | | | |

**Supplementary Table 5. Employment to population ratio, employment status change and all-cause mortality, Finland, 1989-1996 & 1997-2007**

|  | 1989-1996 | | | | | | | | | | | | 1997-2007 | | | | | | | | | | | |
| --- | --- | --- | --- | --- | --- | --- | --- | --- | --- | --- | --- | --- | --- | --- | --- | --- | --- | --- | --- | --- | --- | --- | --- | --- |
|  | Males | | | | | | Females | | | | | | Males | | | | | | Females | | | | | |
|  | RR |  | 95%CI | | |  | RR |  | 95%CI | | |  | RR |  | 95%CI | | |  | RR |  | 95%CI | | |  |
|  |  |  |  |  |  |  |  |  |  |  |  |  |  |  |  |  |  |  |  |  |  |  |  |  |
| Employment to pop. ratio | 1.04 | ( | 1.03 | , | 1.03 | ) | 1.02 | ( | 0.99 | , | 1.05 | ) | 1.00 | ( | 0.99 | , | 1.01 | ) | 1.00 | ( | 0.98 | , | 1.02 | ) |
| Age | 1.05 | ( | 1.05 | , | 1.06 | ) | 1.06 | ( | 1.06 | , | 1.06 | ) | 1.05 | ( | 1.05 | , | 1.06 | ) | 1.06 | ( | 1.05 | , | 1.06 | ) |
| Employm. status change |  |  |  |  |  |  |  |  |  |  |  |  |  |  |  |  |  |  |  |  |  |  |  |  |
| *Stable employment* | 1.00 |  |  |  |  |  | 1.00 |  |  |  |  |  | 1.00 |  |  |  |  |  | 1.00 |  |  |  |  |  |
| *Job loss* | 2.11 | ( | 1.97 | , | 1.98 | ) | 1.51 | ( | 1.21 | , | 1.90 | ) | 2.50 | ( | 2.27 | , | 2.76 | ) | 1.70 | ( | 1.50 | , | 1.93 | ) |
| *Newly employed* | 1.89 | ( | 1.78 | , | 1.78 | ) | 1.23 | ( | 1.10 | , | 1.38 | ) | 1.89 | ( | 1.76 | , | 2.03 | ) | 1.34 | ( | 1.22 | , | 1.47 | ) |
| *Long-term unemployed* | 3.37 | ( | 3.13 | , | 3.13 | ) | 2.19 | ( | 1.90 | , | 2.53 | ) | 4.40 | ( | 4.08 | , | 4.75 | ) | 2.67 | ( | 2.32 | , | 3.06 | ) |
| *Out of workforce* | 4.38 | ( | 4.19 | , | 4.19 | ) | 3.77 | ( | 3.67 | , | 3.87 | ) | 5.53 | ( | 5.38 | , | 5.68 | ) | 5.00 | ( | 4.72 | , | 5.29 | ) |
|  |  |  |  |  |  |  |  |  |  |  |  |  |  |  |  |  |  |  |  |  |  |  |  |  |
| *All models include age, region and year fixed effects but estimates are omitted from Table*  *The category 'out of workforce' refers to individuals that were out of the workforce in either year t-1 or t-2* | | | | | | | | | | | | | | | | | | | | | | | | |

**Supplementary Table 6. Interaction between educational level and employment to population ratio, employment status change and all-cause mortality, Finland, 1989-1996 & 1997-2007**

|  | 1989-1996 | | | | | | | | | | |  |  | 1997-2007 | | | | | | | | | | |  |
| --- | --- | --- | --- | --- | --- | --- | --- | --- | --- | --- | --- | --- | --- | --- | --- | --- | --- | --- | --- | --- | --- | --- | --- | --- | --- |
|  | Males | | | | | | Females | | | | | |  | Males | | | | | | Females | | | | | |
|  | RR |  | 95%CI | | |  | RR |  | 95%CI | | |  |  | RR |  | 95%CI | | |  | RR |  | 95%CI | | |  |
|  |  |  |  |  |  |  |  |  |  |  |  |  |  |  |  |  |  |  |  |  |  |  |  |  |  |
| Employment to Population ratio | 0.97 | ( | 0.93 | , | 1.01 | ) | 0.98 | ( | 0.93 | , | 1.04 | ) |  | 1.01 | ( | 0.97 | , | 1.05 | ) | 0.99 | ( | 0.94 | , | 1.04 | ) |
|  |  |  |  |  |  |  |  |  |  |  |  |  |  |  |  |  |  |  |  |  |  |  |  |  |  |
| Educational level |  |  |  |  |  |  |  |  |  |  |  |  |  |  |  |  |  |  |  |  |  |  |  |  |  |
| Upper tertiary | 1.00 |  |  |  |  |  | 1.00 |  |  |  |  |  |  | 1.00 |  |  |  |  |  | 1.00 |  |  |  |  |  |
| Lower tertiary | 0.95 | ( | 0.29 | , | 3.14 | ) | 0.57 | ( | 0.19 | , | 1.73 | ) |  | 2.69 | ( | 1.78 | , | 4.06 | ) | 1.51 | ( | 0.59 | , | 3.88 | ) |
| Secondary | 1.47 | ( | 0.83 | , | 2.59 | ) | 1.28 | ( | 0.51 | , | 3.24 | ) |  | 3.73 | ( | 2.46 | , | 5.65 | ) | 3.36 | ( | 1.91 | , | 5.93 | ) |
| Basic or unknown | 4.71 | ( | 2.66 | , | 8.37 | ) | 4.52 | ( | 1.84 | , | 11.11 | ) |  | 15.71 | ( | 9.85 | , | 25.07 | ) | 16.62 | ( | 9.66 | , | 28.61 | ) |
|  |  |  |  |  |  |  |  |  |  |  |  |  |  |  |  |  |  |  |  |  |  |  |  |  |  |
| Lower tertiary *unemp. rate | 1.05 | ( | 0.97 | , | 1.14 | ) | 1.02 | ( | 0.96 | , | 1.10 | ) |  | 1.00 | ( | 0.96 | , | 1.04 | ) | 1.02 | ( | 0.94 | , | 1.11 | ) |
| Secondary*unemp. rate | 1.09 | ( | 1.05 | , | 1.13 | ) | 1.03 | ( | 0.96 | , | 1.10 | ) |  | 1.00 | ( | 0.96 | , | 1.04 | ) | 1.01 | ( | 0.95 | , | 1.07 | ) |
| Basic/unknown*unemp. rate | 1.07 | ( | 1.03 | , | 1.11 | ) | 1.04 | ( | 0.98 | , | 1.11 | ) |  | 0.98 | ( | 0.95 | , | 1.02 | ) | 1.01 | ( | 0.95 | , | 1.07 | ) |
|  |  |  |  |  |  |  |  |  |  |  |  |  |  |  |  |  |  |  |  |  |  |  |  |  |  |
|  |  |  |  |  |  |  |  |  |  |  |  |  |  |  |  |  |  |  |  |  |  |  |  |  |  |
| *All models include age, employment status change, region, year fixed-effects, and interactions of educational level with all variables* | | | | | | | | | | | | | | | | | | | | | | | | | |

**Supplementary Figure 1. Harmonized unemployment rate (out of total labour force), 1989-2014, Finland**

*Source: Organization for Economic Cooperation and Development*[*^19^*](#_ENREF_19)

**Supplementary Figure 2. Unemployment rate (15-74 years) in 10 regions with largest populations from 1989 to 2007 in Finland**

**Supplementary Figure 3. Impact of one-point increase in regional unemployment rate on cause-specific mortality, Finland, 1989-2007**

*All models include age, employment status change, region and year fixed-effects*

**Supplementary Figure 4. Hodrick-Prescott Filter**-**Detrended regional unemployment rate and cause-specific mortality, Finland, 1989-2007**

*All models include age, employment status change, region and year fixed-effects*

**Supplementary Figure 5. Hodrick-Prescott Filter**-**Detrended regional unemployment rate and mortality by education, Finland, men, 1989-1996**

*All models include age, employment status change, region and year fixed-effects, as well as interactions of educational level with all variables*
